# Electrical Properties of Composite Materials with Electric Field-Assisted Alignment of Nanocarbon Fillers

**DOI:** 10.1186/s11671-017-2244-0

**Published:** 2017-07-28

**Authors:** Olena Yakovenko, Ludmila Matzui, Ganna Danylova, Victor Zadorozhnii, Ludmila Vovchenko, Yulia Perets, Oleksandra Lazarenko

**Affiliations:** Physics Department, Taras Shevchenko National University of Kyiv, Volodymyrska Str., 64/13, Kyiv, 01601 Ukraine

**Keywords:** Composites, Carbon nanotubes, Graphite nanoplatelets, Electric field-induced alignment, Depolarization factor, Electric conductivity, Dynamic percolation, 72.80.Tm82.35.Np81.05.uf81.07.De77.22.Ej87.50.Rr64.60.ah64.60.aq

## Abstract

The article reports about electric field-induced alignment of the carbon nanoparticles embedded in epoxy matrix. Optical microscopy was performed to consider the effect of the electric field magnitude and configuration, filler morphology, and aspect ratio on alignment process. Characteristic time of aligned network formation was compared with modeling predictions. Carbon nanotube and graphite nanoplatelet rotation time was estimated using an analytical model based on effective medium approach. Different depolarization factor was applied according to the geometries of the particle and electric field.

Solid nanocomposites were fabricated by using AC electric field. We have investigated concentration dependence of electrical conductivity of graphite nanoplatelets/epoxy composites using two-probe technique. It was established that the electrical properties of composites with random and aligned filler distribution are differ by conductivity value at certain filler content and distinguish by a form of concentration dependence of conductivity for fillers with different morphology. These differences were explained in terms of the dynamic percolation and formation of various conductive networks: chained in case of graphite nanoplatelets and crossed framework in case of carbon nanotubes filler.

## Background

Tailorable properties provide an application of conductive carbon-based composite materials in many industries as electronic and constructional components: in microelectronics, electrostatic dissipation, at electromagnetic shields fabrication, for aircraft structures, etc. [1–4]. They do not corrode like metals but possess appropriate strength, weight, and wide range of conductivity values due to a variety of employed fillers.

Non-spherical particles are more favorable as composite fillers from the perspective of improving electrical conductivity of composite [5–7]. This is due to lower values of packing factor at increase of particles aspect ratio [8], which is a parameter of statistical percolation model. Therefore, the development of composites with carbon nanotubes (CNTs) and graphite nanoplatelets (GNPs), which are characterized by high values of aspect ratio (10^1^–10^4^) [9], as fillers, is a very promising direction. Besides geometrical anisotropy, CNTs and GNPs distinguish by the anisotropy of physical properties. But at random distribution of filler at composite the anisotropy of individual filler particle is compensated. Besides, at random filler distribution, much of it is concentrated in the so-called “blank” branches of a conductive network which are disjoint from the overall network. These losses are particularly considerable at low content of filler in the composite.

Recomposing of filler and its specific spatial distribution allows obtaining composites with low percolation threshold which reduces the cost of material. The most popular methods of preparation of composites with anisotropic filler distribution are exposure of liquid composite mixture to electromagnetic field and application of mechanical stress. Among disadvantages of filler alignment method by rolling, shearing stress is possible breaking and destruction of carbon nanoparticles under such exposure [10]. Magnetic field-induced alignment requires addition of magnetic components to the composite [11]. Thus, electric field-assisted alignment of the filler in composite is the most promising method of anisotropic composite formation from the standpoint of many research groups [12, 13].

But the overwhelming majority of the presented works about electric field-assisted alignment are devoted to carbon nanotubes embedded in polymer matrix [14–18]. The effect of the morphology of the filler particles on the process of alignment is very poorly developed in both theoretical and experimental studies [19–21]. The aim of this study was to investigate the influence of filler morphology on the process of aligned composite formation and to identify and explain differences of concentration dependence of the electrical conductivity of the composites with random and aligned GNP distribution.

## Methods

### Materials

Based on epoxy resin Larit 285 (Lange Ritter GmbH, Germany), composite materials were fabricated and investigated in this study. In the initial state, this polymer is two-component and consists of liquid epoxy and appropriate hardener H 285. Low viscosity of the used resin (600 ÷ 900 mPa × s at 25 °С) and hardener (50 ÷ 100 mPa × s at 25 °С) allows using the impact of an external electric field for the manufacture of composite materials on their basis.

The following materials were used as fillers for the fabricated composite systems:

- Multiwall carbon nanotubes (MWCNTs) (Cheap Tubes Ins, USA);
- Graphite nanoplatelets (GNPs).

GNPs were obtained by ultrasonic dispersing (in acetone medium for 3 h) of thermally exfoliated graphite which is a resulting product of deep thermo-chemical treatment of dispersed graphite. The process of GNP manufacturing is described in detail in [22].

Table 1 contains parameters of the used fillers. Their dimensions and shape were estimated by using AFM, SEM, and optical microscopy in papers of our research group [23, 24]. GNP and MWCNT particles distinguish by shape, size and, therefore, an aspect ratio. Table 1 contents characteristic parameters for the largest, smallest, and average particles. The particles are marked “max,” “min,” and “aver” by the values of their aspect ratio. Evaluating mass of the particles suggested that density *ρ*(MWCNT) = 1.8 g/cm^3^ [25], *ρ*(GNPs) = 2.23 g/cm^3^, as the density of monocrystalline graphite.

**Table 1:** Parameters of carbon nanoparticles

Particle sign	Shape	Length, m	Outer radius, m	Inner radius, m	Thickness, m	Aspect ratio	Mass, kg
GNP_min_	Spheroid, Ellipsoid, Disk	–	0.2 × 10^−6^	–	5 × 10^−9^	80	1.4 × 10^−18^
GNP_max_			30 × 10^−6^		65 × 10^−9^	923	4.1 × 10^−16^
GNP_aver_			5 × 10^−6^		30 × 10^−9^	333	5.3 × 10^−18^
CNT_max_	Ellipsoid, Cylinder	10 × 10^−6^	5 × 10^−9^	2 × 10^−9^	–	1000	1.19 × 10^−18^
CNT_min_			15 × 10^−9^	6 × 10^−9^		333	10.68 × 10^−18^

### Composite Fabrication

Composite samples that were prepared contained different concentrations of GNPs as a filler (0.05, 0.5, 0.7, 1, 2, 3, 4, 5 wt%). One type of samples had random distribution of the filler, and in another one, GNPs were aligned by external electric field.

Method of composite systems preparation was as follows. At first, required amount of carbon filler was poured into pre-dissolved polymer matrix and mixed mechanically. After that, the mixture was exposed to ultrasonic dispersing for a better distribution of filler in epoxy matrix. Dispersion was carried out in ultrasonic bath Вaku-9050 with frequency of 40 kHz and maximum output electric power of 50 W. The composite mixture was exposed to ultrasonic action for 30 min at 50 W, after that hardener Н 285 was added to the resulting composite mixture in 100/40 ratio by mass to the weight of Larit 285.

Eventually, for composites with aligned filler distribution fabrication, a part of obtained composite mixture was poured into plastic mold which was placed between the plates of capacitor. AC voltage with frequency of 15 kHz and magnitude of 2000 V was applied to the plates. High-voltage source with the ability to generate AC voltage at 15 kHz frequency and magnitude in the range up to 2000 V or DC voltage with magnitude in the range up to 2000 V was used as a source of electric field. The value of the magnitude of electric field was controlled by universal voltmeter В7-16А.

Under choosing the AC electric field frequency, we kept in mind two points: (1) the frequency should be high enough that the carbon nanoparticles alignment time would be the time of epoxy hardening; (2) the frequency should be low enough to observe the dynamics of nanoparticles alignment in electric field. Keeping in mind these considerations, we have carried out composites formation at the frequency of 15 kHz.

The other part of the composite mixture was left without the influence of an external electric field.

After holding at room temperature, molds with composite samples were subjected to heat treatment at stepwise increasing temperature from 40 to 80 °C, which increased on 10 °C hourly. This was done to complete polymerization process of composites.

### Optical Microscopy

Investigation of the character of carbon fillers distribution in epoxy matrix under electric field treatment was performed for composites with the content of carbon filler of 0.05 wt%. This was done with a stereoscopic optical microscope МBS-1 equipped with digital camera Etrek DCM-510. This setup provided the opportunity of observation online of the liquid epoxy with dispersed carbon nanoparticles under electric field influence. The configuration of experiment is described in detail in [26, 27]. A series of optical observations of the MWCNTs/− and GNPs/Larit 285 composites was conducted in real time supplying electrodes with AC voltage of 15 kHz or DC voltage and changing the value of electric field magnitude.

### Electrical Properties Measurement

Electrical conductivity of the investigated composites was measured by standard two-probe method in DC mode at room temperature with a limit of measurement of electrical resistance of 10^10^ ohm. Higher than 10^10^ ohm, resistances were measured using teraommetr Е6–13. Samples for measurements were prepared in the form of regular parallelepipeds with the dimensions 5.0 × 4.0 × 4.0 mm^3^.

### Modeling

Equations by which characteristic time of carbon particle rotation under electric field action was estimated were solved using a mathematical package Maple 13.

## Results and Discussion

### Optical Observations

**Fig. 1:**
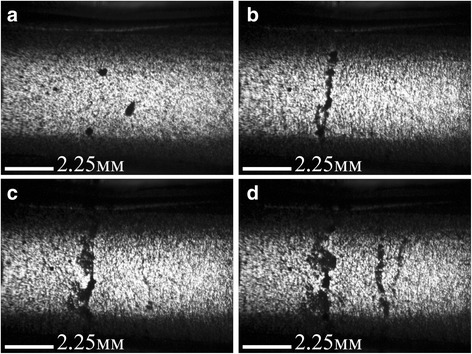
Optical images of electrical “breakdown” formation in 0.05 wt% GNPs/Larit 285 composite under AC electric field action of strength of 36 kV/m, frequency of 15 kHz (embedded electrodes): **a**—before electric field action; **b**—after 100 s; **c**—after 140 s; **d**—after 160 s of electric field action. Image size 10.8 × 8.0 mm^2^

**Fig. 2:**
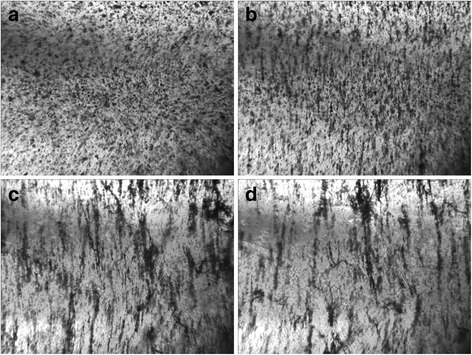
Optical images of 0.05 wt% GNPs/Larit 285 composite under AC electric field action (frequency of 15 kHz, strength of 167 kV/m) (in capacitor): **а—**before electric field action; **b**—after 12 min; **c**—after 26 min; **d**—after 60 min of electric field action. Image size 10.8 × 8.0 mm^2^

**Fig. 3:**
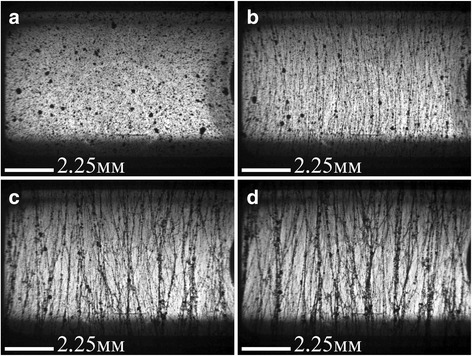
Optical images of 0.05 wt.% MWCNTs/Larit 285 composite under AC electric field action (frequency of 15 kHz, strength of 83.3 kV/m) (embedded electrodes): **a** – before electric field action; **b** – after 12 min; **c** – after 26 min; **d** – after 60 min of electric field action. Image size 10,8 × 8,0 mm^2^ [26]

**Fig. 4:**
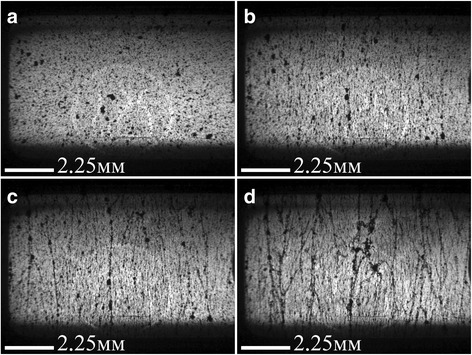
Optical images of 0.05 wt% MWCNTs/Larit 285 composite under AC electric field action (frequency of 15 kHz, strength of 50 kV/m) (embedded electrodes): **а**—before electric field action; **b**—after 12 min; **c**—after 26 min; **d**—after 60 min of electric field action. Image size 10.8 × 8.0 mm^2^ [26]

The following figures show optical photos of the surface of composite materials GNPs/Larit 285 (Figs. 1 and 2) and MWCNTs/Larit 285 (Figs. 3 and 4) [26] of low filler content (0.05 wt%) at the action of AC electric field.

Processed with AC electric field, composites became more transparent with increasing time of electric field action due to filler movement in the direction of power lines of an external electric field. It was also observed that with increasing time of electric field action, the aligned structures of carbon filler broaden and some clearances emerge between them. This may be due to Van der Waals interactions of carbon nanoparticles. In this manner, chains of nanocarbon filler of specified distribution are forming in composite.

As seen in Fig. 1, for GNPs/Larit 285 composites in embedded electrodes configuration, AC electric field action manifests itself in creation of the main conductive path, which closes the supply with external voltage electrodes and leads to a high amplitude current flow through the composite sample. Therefore, alignment of GNPs in Larit 285 was performed by the experimental set when the composite is placed between plates of capacitor. Figure 2 shows that such a configuration of experimental setup in which the current flow through the sample is impossible allows obtaining consistently aligned chains of GNPs in polymer matrix.

In case the of MWCNTs/Larit 285 composites treatment with AC electric field, a formation of elongation in the direction of the electric field chains was observed as well. Distinct chains become thicker when time of AC field exposure increases. But characteristic time of aligned chain formation in MWCNTs/epoxy composite is minutes while in GNPs/epoxy composite is seconds in embedded electrodes configuration. Moreover, due to GNP shape and size, aligned filler distribution in GNPs/epoxy composites can be formed at less magnitudes of AC electric field than in MWCNTs/epoxy composites. But on the other hand due to GNPs mobility because of its shape and size in comparison with MWCNTs, the formation of bulk composites with GNP filler is complicated. Aligned network of GNPs tends to destroy shortly after switching off the electric field if the composite has not polymerized completely.

When considering all optical images of aligned network under AC electric field formation in GNPs/− and MWCNTs/epoxy composites, we can conclude that under equal conditions when changing only the type of carbon filler, the aligned network is formed faster in the GNPs/epoxy composite. In the case of MWCNTs, their tendency to agglomeration prevents effective alignment in the direction of the applied electric field. It should be noted that DC field is not effective for formation of aligned network in composite [26].

To explain the observation by optical microscopy peculiarities of electric field-assisted alignment of the filler of different morphology, a characteristic time of carbon nanoparticle rotation in epoxy matrix under AC electric field action was theoretically estimated.

### Modeling of Carbon Particle Alignment in Viscous Medium

The mechanism of composite with a specified spatial distribution of filler formation method is that every carbon nanoparticle which is embedded in dielectric matrix undergoes polarization under AC electric field action due to the polarization of the interface between the polymer and the particle. Generally, polarizing moment and the electric field vector are noncollinear due to anisotropy of the nanoparticles. Therefore, when the electric field is activated, a torque which leads to carbon nanoparticle rotation in the direction of the field occurs. It is known that the rotational motion of a particle in this case is described by the following equation [16]:

1 $$ I\frac{d^2\varTheta }{dt^2}+{T}_{\eta }+{T}_{\mathrm{align}}=0 $$

where *I* is the moment of inertia of carbon nanoparticle; *Θ* is the angle between the particle and the electric field direction; *T*_*η*_ is a damping torque; ${T}_{\mathrm{align}}\approx \left[\overrightarrow{\mu}\times \overrightarrow{E}\right]$ is field-induced torque; $\overrightarrow{\mu}=f\left(\varepsilon, {\sigma}_1,{\sigma}_2,v\right)$ is the polarizing moment which depends on the values of dielectric constant (*ε*) and conductivity (*σ*_1_, *σ*_2_) of nanoparticle and matrix; *v* = *f*(*m*, *l*, *d*) is the volume of carbon nanoparticle which depends on its weight (*m*) and dimensions (*l*, *d*).

Generally, polarizing moment $\overrightarrow{\mu}$ is proportional to the external field $\overrightarrow{E}$ and particle’s volume *ν* and is determined by the formula [28]:

$$ \overrightarrow{\mu}={\varepsilon}_0{\varepsilon}_m\beta \nu \overrightarrow{E} $$

where *ε*_0_ is the permittivity of free space, *ε*_*m*_ is a dielectric constant of the matrix, *β* is a dimensionless parameter which, in particular, depends on the shape of inclusion. In [28], formulas of *β* for an ideal conductive disc and cylinder are given:

$$ {\beta}_{\perp}^{\mathrm{disk}}=\frac{\sigma_p-{\sigma}_m}{\sigma_p},\kern1em {\beta}_{II}^{\mathrm{disk}}=\frac{\sigma_p-{\sigma}_m}{\sigma_m}; $$

$$ {\beta}_{\perp}^{\mathrm{cylinder}}=\frac{2\left({\sigma}_p-{\sigma}_m\right)}{\sigma_p+{\sigma}_m},\kern1em {\beta}_{II}^{\mathrm{cylinder}}=\frac{\sigma_p-{\sigma}_m}{\sigma_m}. $$

It follows from these dependencies that ${\overrightarrow{\mu}}_{II}\ne {\overrightarrow{\mu}}_{\perp }$ (*II* means codirection of the long axis of the particle and of the field direction, ⊥—perpendicularity). Thus, for GNPs and MWCNTs, ${\overrightarrow{\mu}}_{II}>{\overrightarrow{\mu}}_{\perp }$ because of their shape and properties.

To evaluate the characteristic time of carbon particle rotation under electric field action and its alignment by the direction of the field Eq. (1) with the initial conditions *Θ*(*t* = 0) = *Θ*_0_, $\frac{d\varTheta }{dt}\left(t=0\right)=0$ were solved. According to [16], terms in the main equation of movement are as follows:

$$ {T}_{\eta }=8\pi \eta \nu \frac{d\varTheta }{dt}, $$

$$ {T}_{\mathrm{align}}=\frac{1}{4}{\nu \varepsilon}_m\operatorname{Re}\left[{\alpha}^{\ast}\right]{E}^2 Sin2\varTheta, \pm $$

where ${\alpha}^{\ast }=\left({\left({\varepsilon}_p^{\ast }-{\varepsilon}_m^{\ast}\right)}^2\right)/\left(\left[{\varepsilon}_m^{\ast }+\left({\varepsilon}_p^{\ast }-{\varepsilon}_m^{\ast}\right){L}_x\right]\left({\varepsilon}_p^{\ast }+{\varepsilon}_m^{\ast}\right)\right)$ is a polarizability, ${\varepsilon}_{m,p}^{\ast }={\varepsilon}_{m,p}-j\frac{\sigma_{m,p}}{\omega }$, and *ε*_*m* , *p*_ , *σ*_*m* , *p*_ are dielectric constant and conductivity of the medium and particle, *ω* = 2*πf*, *f*—electric field frequency.

**Fig. 5:**
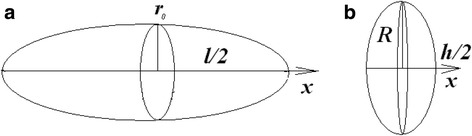
Schematic images of carbon nanotube (**a**) and graphite nanoplatelet (**b**)

To determine the depolarization factor of carbon nanotube and graphite nanoplatelet, they must be considered as the particles of certain shape (Fig. 5). MWCNT can be considered as ellipsoid only. The ellipticity of the MWCNT in ellipsoid approximation is $e=\sqrt{1-{\left(2{r}_0/l\right)}^2}$. GNP can be considered as spheroid or as ellipsoid. The ellipticity of the GNP in ellipsoid approximation is $e=\sqrt{1-{\left(h/2R\right)}^2}$ and in spheroid approximation is $e=\left(2R/h\right)\sqrt{1-{\left(h/2R\right)}^2}$. Then expressions of depolarization factor take the following form [28].

For the ellipsoid:

$$ {L}_x=\frac{1-{e}^2}{e^3}\left( Arth\kern0.5em e-e\right). $$

For the spheroid:

$$ {L}_x=\frac{1+{e}^2}{e^3}\left(e- \arctan \kern0.5em e\right),\kern2.5em {L}_x+2{L}_R=1. $$

In the above equations, *L*_*x*_ is a depolarization factor if the external field is applied along the *x*-axis (as in Fig. 5), *L*_*R*_ is a depolarization factor if the external field is applied along the radius of GNP.

Expending the depolarization factors in series, the above expressions take the following form.

For the ellipsoidal MWCNT:

2 $$ {L}_x=\frac{4{r}_0^2}{l^2}\left[ \ln \left(\frac{l}{r_0}\right)-1\right] $$

For the ellipsoidal GNP:

3 $$ {L}_x=\frac{h^2}{4{R}^2}\left[ \ln \left(\frac{4R}{h}\right)-1\right] $$

For spherical GNP:

4 $$ {L}_x\approx 1-\frac{h\left(8\pi {R}^2-16hR+3\pi {h}^2\right)}{32{R}^3} $$

5 $$ {L}_R\approx \frac{h\left(8\pi {R}^2-16hR+3\pi {h}^2\right)}{64{R}^3} $$

In addition, to highlight the peculiarities of particles morphology, CNT volume was asked as the volume of a hollow cylinder $\nu =\pi l\left({r}_0^2-{r}_i^2\right)$, while GNP volume was asked as the volume of a disk *ν* = *πR* ^2^ *h*.

Carbon nanotube moment of inertia was taken as $I=\frac{ml^2}{12}$—the moment of inertia of straight thin rod with the length of *l* and mass of *m*, the rotation axis is perpendicular to the rod and passes through its center of mass. Graphite nanoplatelet moment of inertia was taken as $I=\frac{mR^2}{2}$—the moment of inertia of disk, the radius of which is *R*, and mass of *m*, disk is rotating about the perpendicular to its plane axis.

Then using all the foregoing approach and setting numerical parameters, calculations of the change of inclination angle of particles of different morphology relative to the field direction with time of AC electric field treatment were carried out.

Basing on paper [29] where experimental concentration dependencies of real and complex parts of dielectric permittivity of the composites with fine graphite and carbon nanotubes were described by the Nielsen formula and equations.

$$ {\varepsilon}_{CNT}^{\ast }=62.2\hbox{--} 12.4\times i,\kern1em {\varepsilon}_c^{\ast }=34.3\hbox{--} 13.4\times i $$

are given, in our calculations for GNP *ε*_*p*(*GNP*)_=34.3, and for MWCNT, *ε*_*p*(*CNT*)_=62.2 was taken.

Geometrical parameters of the particles were taken from Table 1. Concerning other used numerical parameters, it was put that *ε*_0_=8.85 × 10^−12^ F/m, *η* = 0.75 Pa × s, *f* = 15 kHz, *ε*_*m*_=2.8*ε*_0_ [30], *σ*_*m*_=10^−6^ Sm/m [16]. Conductivity of individual carbon particles was taken as *σ*_*p*(*CNT*)_=10^5^ Sm/m [31], *σ*_*p*(*GNP*)_=10^5^ Sm/m [32, 33]_._

**Fig. 6:**
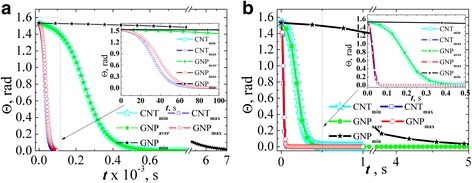
The evolution of the angle of carbon filler particles inclination relative to the field direction estimated in ellipsoids approximation for AC electric field strength 1 kV/m (**a**) and 36 kV/m (**b**)

Figure 6 shows inclination angle of the particle relative to the direction of the applied field dependence of the field action time when *L*_*x*_ was evaluated by Eqs. (2) and (3). The results were found for two values of field strength: 1 kV/m (Fig. 6а) and 36 kV/m (Fig. 6b) supposing that the particle is almost completely disordered at the initial time (*Θ*(*t* = 0) = *π*/2.05).

There has been a clear correlation between the aspect ratio of the particles and the time of alignment by the electric field. Namely, maximum time for alignment is for particles with the lowest aspect ratio (GNP_min_). For GNP_max_ and CNT_max_, alignment time is almost equal, and equal time of alignment is for GNP_aver_ and CNT_min_.

**Fig. 7:**
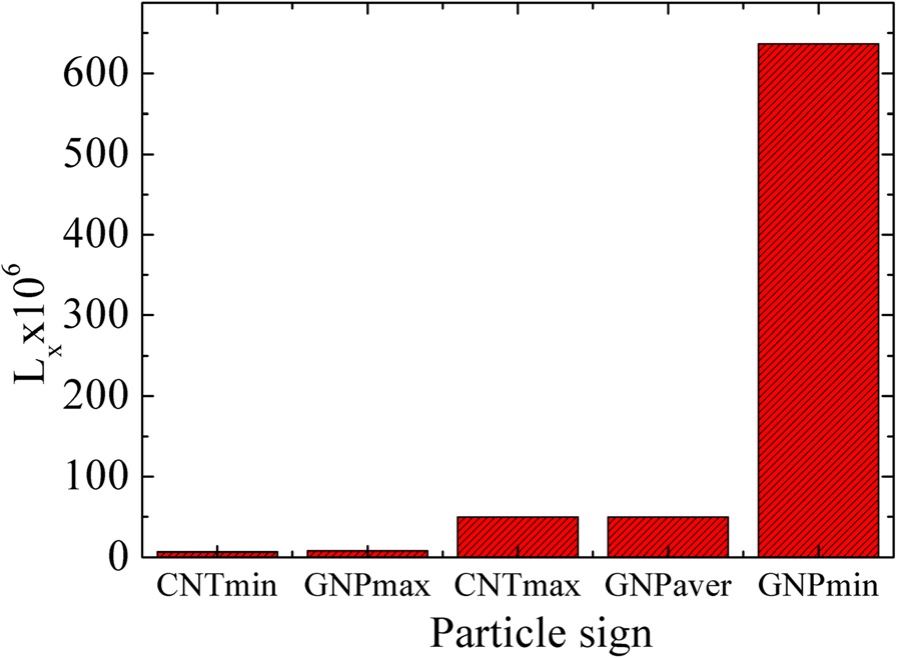
Depolarization factor for GNPs and MWCNTs evaluated by Eqs. (2) and (3)

Figure 7 shows depolarization factor values which was estimated by Eqs. (2) and (3) for GNPs and MWCNTs. *L*_*x*_ is a geometrical factor and depends not on the absolute values of semi axes of the simulated ellipsoids but on their ratio. Thus, *L*_*x*_ is a direct function of the particle aspect ratio.

That is, the depolarization factor is the main parameter of the problem. Since its expression depends on the particle morphology and size a characteristic time of GNPs and MWCNTs rotation differ.

**Fig. 8:**
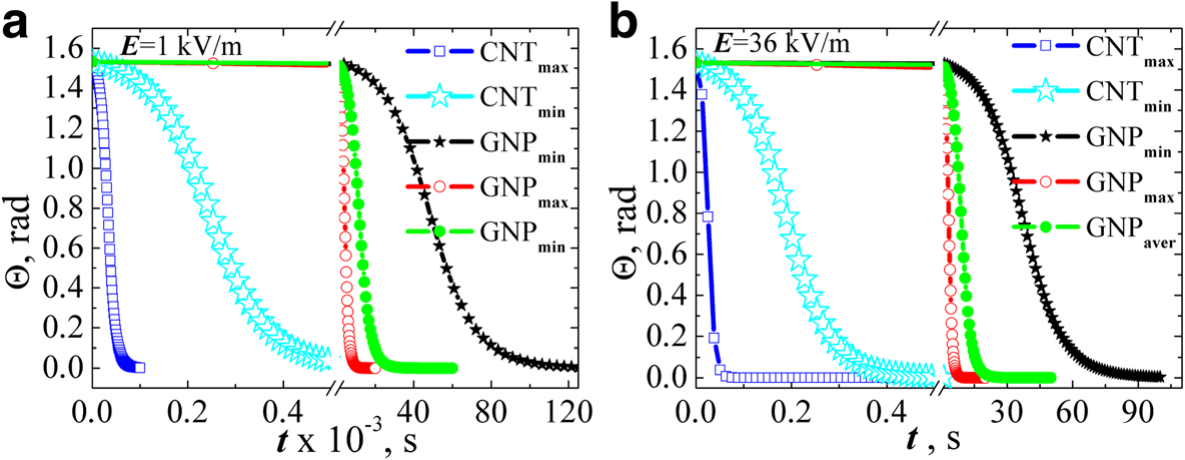
The evolution of the angle of carbon filler particles inclination relative to the field direction estimated in ellipsoids approximation for MWCNTs and in spheroid approximation for GNPs at AC electric field strength 1 kV/m (**a**) and 36 kV/m (**b**)

Figure 8 contents analogous to Fig. 6 dependences of inclination angle of the GNPs relative to the direction of the applied field on the field action time when *L*_*R*_ was evaluated by Eq. (5). The results were found for two values of field strength: 1 kV/m (Fig. 8а) and 36 kV/m (Fig. 8b) supposing that the particles are almost completely disordered at the initial time (*Θ*(*t* = 0) = *π*/2.05). For comparison, the results of calculations of the angle dependence for MWCNTs (*L*_*x*_ was evaluated by Eq. (2)) are introduced at the same graphs.

**Fig. 9:**
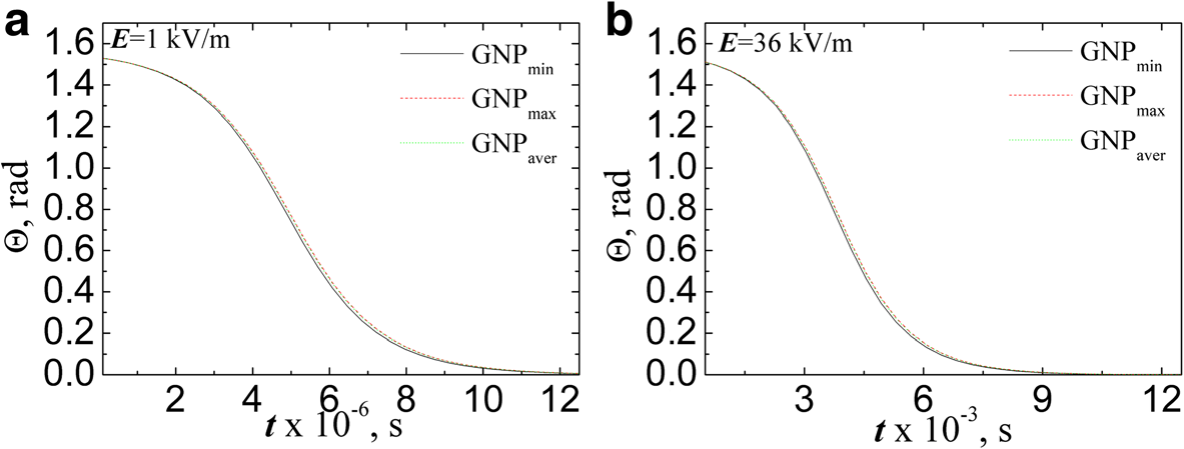
The evolution of the angle of GNPs inclination relative to the field direction estimated in spheroid approximation (*L*_*x*_ was taken by the Eq. (4)) at AC electric field strength 1 kV/m (**a**) and 36 kV/m (**b**)

Figure 9 shows dependences of inclination angle of the GNPs relative to the direction of the applied field on the field action time in approximation that GNP is a spheroid with depolarization factor (4). Evaluation was performed for AC electric field strength 1 kV/m (Fig. 9а) and 36 kV/m (Fig. 9b) supposing that at initial time the particle is approximately disordered (*Θ*(*t* = 0) = *π*/2.05).

From the analysis presented in Fig. 9 data, the following conclusions were made: firstly, if we assume that the electric field axis is codirected with the *x*-axis of GNP, the rotation time increases significantly compared with the calculation in case of the field axis and GNP radius codirection. And this behavior is irrespective of the GNPs aspect ratio. Secondly, the course of the dependence is minimally different for the particles with different aspect ratios, and at a certain point of process time, more aligned particle is the particle with minimum aspect ratio GNP_min_ while GNP_aver_ and GNP_max_ angles coincide. This behavior is due to the value of depolarizing factor which is close to 1 for the above cases.

Thus, the estimation have shown that the rotation time of carbon particles under AC electric field action depends on their morphology and aspect ratio. Note that the model considers one particle embedded in polymer matrix while in composite, we have an ensemble of particles with different initial angles of inclination. That is one of the reasons why real characteristic time of the whole network formation may be very different from the estimated time.

Besides, it is complicated to achieve such a distribution of MWCNTs in composite mixture where each individual tube is entangled. It is known that carbon nanotubes tend to tangle due to the interaction of their surfaces. Therefore, the real time of MWCNTs in composite mixture alignment is significantly higher than it was theoretically estimated. Besides, the viscosity of composite mixture with the same content of MWCNTs is higher than the viscosity of composite mixture with GNPs. All these factors prevent such a rapid alignment of MWCNTs in composite as it was estimated.

### Electrical Properties of Solid Composites

**Fig. 10:**
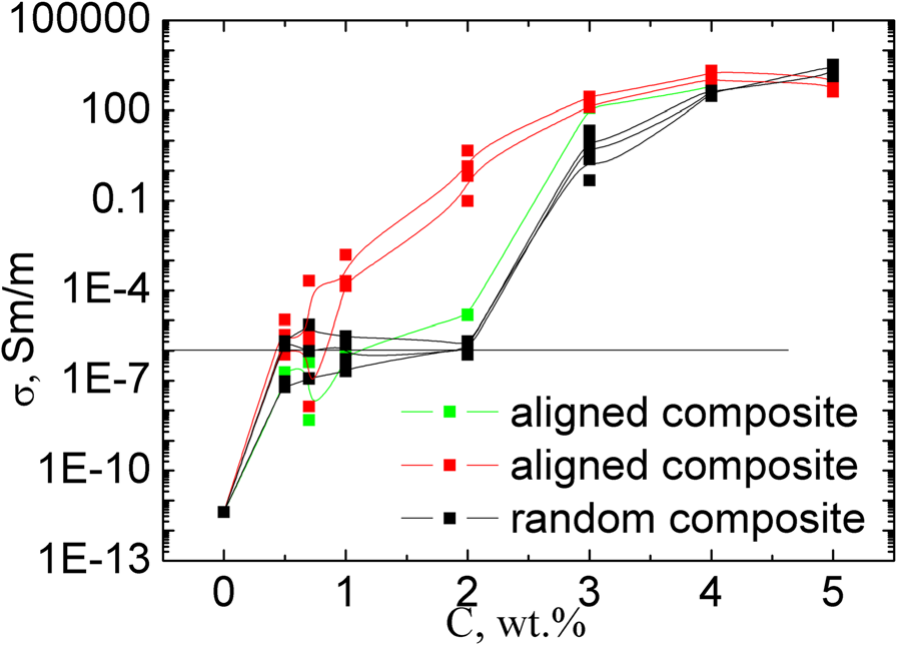
Concentration dependence of electrical conductivity of GNPs/Larit 285 composites with aligned and with random filler distribution in logarithmic scale

**Fig. 11:**
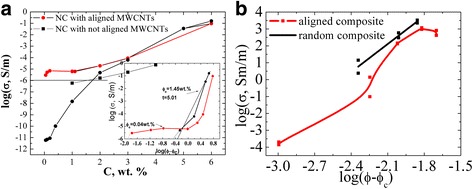
Concentration dependence of conductivity of MWCNTs/Larit 285 (**а**) [27] and GNPs/Larit 285 (**b**) in log-log scale

Within the study, values of resistivity of the prepared GNPs/Larit 285 composites with aligned and with random filler distribution in epoxy matrix were experimentally found. Figure 10 presents the concentration dependences of electrical conductivity of GNPs/Larit 285 composites with aligned and with random filler distribution. Electrical properties of the composite samples were investigated in longitudinal and perpendicular to the applied at composite fabrication electric field direction. Figure 11 presents concentration dependences of conductivity for MWCNTs/Larit 285 (а) [27] and GNPs/Larit 285 (b) composites in log-log scale.

The lowest values of the conductivity *σ* correspond to the samples of epoxy resin without filler (conductivity of 10^−11^ Sm/m). With GNPs adding to the composite, its conductivity increases and reaches the value of *σ* = 10^−6^ Sm/m (this value is considered as percolation level) at GNPs content *с* in composite of *φ*_*с*_ = 2 wt% for the composites with random filler distribution. For the GNPs/Larit 285 composites with aligned filler distribution, percolation concentration is of *φ*_*с*_ = 0.84 wt%. Thus, materials of the identical composition are characterized by different values of percolation threshold depending on the method of preparation and filler distribution in the polymer matrix (aligned or random).

Conductivity of GNPs/Larit 285 composites smoothly increases with the increment of GNPs content for both types of the samples while the shape of the dependence is completely different for the MWCNTs/Larit 285 composites obtained by the same method [27] (see Fig. 11). Concentration dependence of conductivity of MWCNTs/Larit 285 composites increases with the increment of MWCNTs content at low filler content until it reaches a plateau, then conductivity increases again. Such a difference of conductivity concentration dependence can be explained by another process of conductive network formation if the fillers are of different morphology. MWCNTs/epoxy composites are characterized by crossed framework structure formation while in GNPs/epoxy composites chained structure appears. GNPs addition to the polymer matrix smoothly increases the number of conductive links in composite. In case of entangled by themselves frame MWCNTs, there is an area where nanotubes addition to the matrix has little effect on its conductivity.

Statistical percolation model operates with probabilities of particles in composite to create a conductive chain at their certain content. As it was shown in our paper, manufactured with electric field treatment, composites become conductive at lower content of carbon filler due to activation of dynamic percolation which is a phenomenon when conductive chain formation is stimulated by external influences at such a content of conductive particles in composite which is not enough for statistical percolation.

It should be noted that the existence of two types of percolation transitions is a characteristic feature of composite materials which are in low-viscosity state during the manufacture [34]. The higher value of percolation concentration cannot be changed by varying the manufacturing conditions of the composite since it is defined by statistical percolation theory. Statistical percolation threshold is defined by the filler type, its aspect ratio, surface state of polymer and filler, wettability, uniformity of filler distribution, and its content in polymer matrix. As we have shown, dynamical percolation threshold can be shifted by activating of filler particle movement in polymer matrix, by electric field action, and, thus, promoting a conductive network formation. The value of dynamic percolation threshold can be changed with method of composite manufacture change. We have established that in case of filler alignment under electric field action, dynamical percolation threshold is defined not only by the above parameters but also by parameters of the applied field and polymer matrix viscosity, filler morphology.

## Conclusions

1. Nanocarbon-polymer composite material with aligned distribution of graphite nanoparticles in epoxy matrix has been produced by exposing to a high-voltage AC electric field. The influence of electric field treatment time, strength, and configuration of electric field on formation of aligned GNPs network in liquid polymer medium was investigated by optical microscopy.
2. It was shown that the influence of AC electric field at composite fabrication process leads to the manifestation of two types of percolation transitions: statistical and dynamic ones. In addition, the aspect ratio of the filler particles and the character of the formation of the conducting cluster, depending on the shape of the particles, determine the shape of the *σ = f(c)* dependence and the critical concentration of both dynamic and statistical percolation thresholds.
3. The effects of the morphology of the filler particles on the process of nanocarbon alignment in polymer matrix under AC electric field have been investigated by estimating of carbon nanotube and graphite nanoplatelet rotation time using an analytical model based on effective medium approach. The theoretical evaluation of characteristic time of carbon nanoparticle of different morphology rotation under AC electric field action have shown that rotation time of carbon nanoparticle is determined by its depolarization factor which in turn depends directly on the aspect ratio of particle.
4. The investigation of concentration dependences of conductivity of composites GNPs/Larit 285 with aligned by AC electric field action filler distribution and random filler distribution in epoxy matrix have shown that under AC electric field action composites, percolation threshold decreases essentially from *ϕ*_*c*_ = 2 wt% for composites with random filler distribution of GNPs to *ϕ*_*c*_ = 0.84 wt% for the obtained under AC electric field action GNPs/Larit 285 composites.

## Abbreviations

AC: Alternative current
AFM: Atomic force microscopy
CNTs: Carbon nanotubes
DC: Direct current
GNPs: Graphite nanoplatelets
MWCNTs: Multiwall carbon nanotubes
SEM: Scanning electron microscopy
